# Non-problem gamblers show the same cognitive distortions while playing slot machines as problem gamblers, with no loss of control and reduced reality control, though – An experimental study on gambling

**DOI:** 10.3389/fpsyg.2023.1175621

**Published:** 2023-05-22

**Authors:** Róbert Krébesz, Dóra Kata Ötvös, Zita Fekete

**Affiliations:** Department of Behavioural Sciences, Faculty of Medicine, University of Debrecen, Debrecen, Hungary

**Keywords:** gambling, cognitive distortions, experiment, slot machine, content analysis

## Abstract

**Background:**

Cognitive distortions can result in maladaptive interpretations of events and maladaptive behavior. In the case of gambling, such distortions can contribute to the maintenance of the disorder. Our current research aimed to conduct an experiment to possibly detect cognitive biases characteristic of persons with gambling addiction in a non-gambling sample of the general population, and to study the effect of “big win” on cognitive distortions.

**Methods:**

A specifically designed and preprogrammed slot machine simulation was carried out, with 90 rounds split into 3 sections. During the simulation every participant verbalized their thoughts and feelings; the verbalizations were recorded. Then a content analysis was conducted to search for indications of cognitive distortions. The sample was separated into two experimental groups: one of the groups experienced the “big wins” in the first section, while the other group had them in the second section of the experiment.

**Results:**

Content analysis revealed numerous cognitive biases. Cognitive distortions usually present in problem gambling were detected in our sample from the general population as well. However, we could not distinguish cognitive biases indicating serious loss of control, or distortion of reality control. A further finding is that early losses provoke the emergence of more cognitive distortions, while early big win leads to more intense loss-chasing in the later stages of gambling.

**Conclusion:**

The appearance of reality-checking uncertainty or loss of control can be alarming for the development of gambling. Losses and big wins can provoke different cognitive distortions, encouraging the person into further gambling behavior.

## 1. Introduction

Cognitive distortions are errors in both cognitive processing and content. These distortions in cognition can result in maladaptive interpretations of the world and its stimuli, and lead to the maintenance of problematic behaviors (Alford and Beck, 1997). In the case of gambling, these distortions are conceived as erroneous or illogical thoughts, assumptions, self-statements, and beliefs that have the potential to lead to more gambling despite losses (Ledgerwood et al., 2020).

As Wildman (1997) points out, gambling contains traces of behavioral patterns that proved to be advantageous for survival during evolutionary development. This is probably also true of the thought processes that influence behavior, and can offer an explanation for the presence of gambling-specific cognitive distortions in the general population (Griffiths, 1994; Toneatto et al., 1997; Goodie and Fortune, 2013).

Many of the findings on distortions in gambling have been derived from observing the speech production of gamblers during gambling. The “thinking-aloud” method is used to investigate gamblers’ thinking content and distortive thoughts through their verbalizations (Gaboury and Ladouceur, 1989). When using this method, subjects are asked to say out loud, uncensored, any emerging thoughts or ideas while playing. The resulting verbal material is audio-recorded and transcribed verbatim for content analysis (Joukhador et al., 2004). These verbal contents may give a broader insight into the individual’s thinking (Brown and Lenneberg, 1954) than questionnaires with predefined questions and scoring methods. Moreover, scales often lack validation on measurement invariance, which makes it difficult to understand the importance of distortions across different levels of severity (Goodie et al., 2019). It is important to point out that cognitive distortions can be present at all levels of cognitive structure, including automatic thoughts and firm beliefs (Burns, 1980; Pössel and Black, 2013). Observing a person’s speech production allows us to study cognitive distortions at the level of automatic thoughts, i.e., maladaptive thoughts during gambling. According to Beck and Haigh (2014) biased thinking processes may reflect the underlying potentially erroneous meanings, interpretations and beliefs.

Different games imply the presence of different cognitive distortions or different profiles of distortions (e.g., Myrseth et al., 2010; Goodie and Fortune, 2013; Clark, 2014; Mathieu et al., 2020). In the following, we present distorted thoughts typical of slot machine gambling.

*Anthropomorphism* appears when attributing intentions, emotions, and motivations (Pancani et al., 2019) to any form of gambling, which may be represented by a machine, a lottery ticket, etc. The *gambler’s fallacy*, another common cognitive distortion in gambling (Croson and Sundali, 2005), is an incorrect belief applying to autocorrelation in a random sequence that shows no autocorrelation in reality (e.g., the latest series can predict what is to come). As Kahneman and Tversky (1972) write (pp. 435), “if the proportions of the two outcomes are to be preserved in short segments, then a long sequence of one outcome must be followed by the other outcome in order to restore the balance.” Besides, gamblers tend to believe that certain aspects are associated with winning, even when there is no causal influence between the accompanying events, for example weather conditions, and gambling (*illusory correlations*) (Toneatto et al., 1997; Toneatto, 2002). Moreover, gamblers tend to mistakenly believe that they have control over the game. The *illusion of control* is the inappropriately high expectancy of personal success despite objective probability. Players often have the misconception that possessing certain skills or knowledge will have a positive influence on the outcome of the game (Langer, 1975). Some gamblers believe that some mysterious force operates in their life (Selzer, 1992). They feel powerful, in control, with no limits (Rosenthal, 1986); this belief is often referred to as *omnipotence*. Furthermore, gamblers tend to overinterpret certain stimuli that have the potential to affect their decision making in gambling, including bodily sensations, omens, intuitions, or unusual events (*overinterpretation of cues*) (Toneatto et al., 1997; Toneatto, 2002). They operate with *flexible attributions*, as they attribute their wins to their own skills, and their losses to external factors (Griffiths and Wood, 2001). Gamblers *selectively recall* their memories of the game: they tend to remember their winning series, and forget about their losses, which can make them feel more successful (Joukhador et al., 2003). They may understand that the outcomes of gambling are the result of chance or randomness; at the same time, they might have beliefs that there are certain means available to influence the outcomes positively. These *superstitious beliefs* may appear in several forms, like using talismans or performing rituals (Toneatto, 1999). These distortions sustain a gambling career; however, losses are inevitably experienced along its course. As losses accumulate, gamblers tend to bet more and more to win back the lost amount (*loss-chasing*) (Lesieur, 1979).

In addition, gambling itself carries structural features that favor the emergence of cognitive biases. Gamblers often have the subjective experience of having won a large amount of money. A special case of this experience is an *early big win*, which – according to definition – occurs at the beginning of a gambler’s career. This leads to false expectations to win, which contributes to continuing gambling despite losses (Weatherly et al., 2004). In other words, big wins can be regarded as reinforcers.

The subjectively perceived chance of potential winning – without minding the reality of losing – can also be facilitated in further ways. The *near-miss effect* describes a losing situation that is falsely perceived by the gambler as close to win (Dores et al., 2020). There are various occurrences of the effect, e.g., in roulette, when the ball lands right next to the number the player bet on; or in lottery, when the player chooses for example number 26 and the draw result is 25 (Kurucz and Kormendi, 2012).

According to Griffiths (1994), there is no clear evidence that cognitive distortions emerging in the verbalizations of gamblers affect the individual’s behavior. However, reinforcement, among others, plays a significant role (Ladouceur and Gaboury, 1988). On the contrary, contemporary models suggest that distortions can predict future involvement in problem gambling and its maintenance (Yakovenko et al., 2016; Goodie et al., 2019). According to the Pathway Model of Blaszczynski and Nower (2002), cognitive distortions are present in all types of gamblers with different etiological backgrounds. As the prevalence and severity of gambling increases, distortive thoughts become more pronounced (Griffiths, 1995), which leads to irrational conclusions about control and probabilities, therefore maintains and exacerbates gambling this way (Delfabbro and Winefeld, 2000).

People tend to simplify their interpretation of the situation when faced with complex situations with difficult-to-predict outcomes. It is also valid for gambling situations, which may explain why some gambling-related cognitive distortions also appear in healthy individuals (Labrador et al., 2020). Moreover, the tendency of simplification of information processing may help us understand why some of these distortions show up even independently of gambling (e.g., anthropomorphism) (Mithen, 1996), while others emerge in the context of gambling (e.g., gambler’s fallacy) (Croson and Sundali, 2005), both in problem and non-problem gamblers.

The prevalence and intensity of cognitive gambling distortions are higher among problem gamblers (Cunningham et al., 2014), and these distortions are accompanied by emotional distress (Ciccarelli et al., 2017). In addition, certain distortions emerge more often among them than among non-problem gamblers, which also points to the role of distortions in developing and maintaining gambling addiction (Labrador et al., 2020). However, there are conflicting results regarding which distortions are less specific to non-problem gamblers (Goodie and Fortune, 2013; Goodie et al., 2019; Labrador et al., 2020).

In our current study, the verbal manifestations of healthy adults were studied during a slot machine simulation, with the help of the “thinking aloud” procedure and deductive content analysis. We explored the participants’ distorted thoughts in a top-down approach. Observing the thoughts of non-problem gamblers may reveal aspects not obtainable by self-reported questionnaires. Targeting the distorted thoughts in non-problem gamblers that have been shown to play a significant role in gambling addiction may provide a way to identify distortions that occur even without a gambling history. In addition, the inclusion of big wins may provide an opportunity to investigate whether these reinforcers influence cognitive distortions in non-problem gamblers.

Thus, our goals were (a) to study the possible presence of cognitive biases – characteristic of problem gamblers – in the automatic thoughts of non-gambling healthy adults, while they took part in a slot-machine simulation; and (b) to map the effect of “big wins” (e.g., significant reinforcement) on the appearance of cognitive biases.

## 2. Materials and methods

### 2.1. Participants

A non-probability sampling method was used, where convenient sampling was applied. Subjects were recruited via advertisements containing a brief study description and the inclusion criteria. No honoraria were offered to participants. The advertisement was distributed online via email to members of the researchers’ mailing lists. Altogether 14 subjects were enrolled in the study this way. The inclusion criteria were the following: at least 18 years of age, no gambling or other psychiatric disorder, including drug or alcohol abuse in the medical history and being a non-gambler. We relied on self-reported information when screening participants for the above inclusion criteria, which all of them met.

### 2.2. Procedure

#### 2.2.1. Slot machine simulation

A computerized three-wheel slot machine was developed with three main interfaces:

1. Instruction page: description of the simulation and a call to subjects to verbalize their thoughts and feelings while playing (for the detailed instructions see Supplementary Material A).
2. Wheel of fortune: apparently chose an initial stake of 5,000 credits at random.

Slot machine: 90 spins (three sets of 30 spins/set) with the well-known “fruit” game; a bet cost a standard of 100 credits.

The wheel of fortune and the 90 spins had preprogrammed outcomes. This ensured that subjects participated in the study under the same conditions (for the detailed description of the simulation see Supplementary Material B).

#### 2.2.2. Design and procedure

An experimental setup with two groups was designed for the study. These groups were formed on the basis of the preprogrammed outcomes of the slot machine simulation:

1. In the Early Big Win Group (EBW Group) a high value win occurred in the first phase of the simulation, i.e., during the first 30 spins.
2. In the Late Big Win Group (LBW Group) the high value win was awarded during the second phase of the simulation.

We defined high value win as fifty times the standard 100 credit bet.

Participants were randomly assigned to EBW and LBW subgroups.

The procedure was completed in four steps:

1. The simulation was preceded by a verbal debriefing: subjects were informed that the study was designed to investigate their thoughts while playing the fruit game. Also, subjects were informed that audio recordings would be made during the game.
2. The next step was the simulation, in which the game was used to map subjects’ thoughts in a gambling situation. In each case, the simulation was conducted in a two-person situation including the subject and an investigator (the first author), and lasted between 1 and 1.5 h. Every subject used the same computer and the study took place in a university office. Participants did not receive any real monetary prizes other than the virtual credits they won in the computerized game.
3. Subjects were informed about the manipulated nature of the program, and signed the post-study informed consent form.
4. Recorded verbal contents were verbatim transcribed, and content analysis was carried out on the transcripts.

#### 2.2.3. Data collection

Sociodemographic data were collected using a questionnaire regarding age, marital status, level of education, self-reported gambling experience, and psychiatric medical history.

Participants were encouraged to freely express their thoughts and feelings during the simulation. When a subject was either deeply engaged in the simulation or was not able to verbalize their thoughts, the investigator asked general questions to facilitate their expressions (e.g., “*What are you thinking right now? How do you feel about the game*?” etc.).

The verbal contents of all 14 participants were analyzed. The mean duration of the recordings was 47.64 min/subject (SD = 17.95; Σ = 667 min; range: 27–90 min). The verbatim transcribed verbal manifestations were merged into one document (51,314 words; 120 pages). The verbal contents of the investigator were not removed but were kept to provide context for the analysis. No software was used for content analysis.

### 2.3. Data analysis

#### 2.3.1. Content analysis

For data analysis, a direct content analysis approach was utilized (Hsieh and Shannon, 2005). Study rigor was ensured following the recommendations by Mays and Pope (1995).

To ensure the reliability of the analysis, two independent coders were employed to code the transcripts, and a third independent investigator inspected the coding, and suggested modifications when necessary. The suggestions of the triangulated investigator were accepted when a 2/3 majority was reached, i.e., when at least one of the two coders agreed with the suggestions of the reviewer. Every modification was recorded. Validity of the analysis was ensured and researcher bias was reduced by applying the above mentioned investigator triangulation (Renz et al., 2018).

Content analysis followed the guidelines by Elo and Kyngas (2008):

First, a comprehensive review of gambling-related cognitive biases was carried out as described above. Then a structured analysis matrix was developed based on the relevant scientific literature. Specified codes were the following (for the definitions of the codes and for examples see Supplementary Material C): Anthropomorphism, Gambler’s fallacy, Illusion of control, Omnipotence, Overinterpretation of cues, Illusory correlations, Flexible attributions, Selective recall, Superstitious beliefs and rituals, Loss-chasing, and Near miss effect.

Data analysis was executed in three steps:

1. After defining the coding categories, two independent coders (DÖ and RK, the first and second authors) read the transcripts individually, and indicated the identified parts of the text that fit the criteria defined in the categorization matrix.
2. The two coders compared their analysis, discussed the ambiguously coded contents and made modifications (in the case of 31 verbal units).
3. The independent reviewer (ZF, the last author) read the coded transcripts and suggested modifications. The reviewer recommended 5 alterations in the coding, out of which 3 were accepted (2/3 majority).

#### 2.3.2. Statistical analysis

The statistical difference between the observed and expected frequencies in the different categories was studied with the help of the Chi-squared test.

### 2.4. Ethics

Ethical approval was granted by the Regional and Institutional Scientific Research Committee of the University of Debrecen (approval number DE RKEB/IKEB: 5641-2021). The study procedures were carried out in accordance with the Declaration of Helsinki. Participants took part in the study voluntarily, their anonymity and the opportunity to end the study at any time were assured. They signed an informed consent form regarding the audio recording made during the simulation and the conditions of the experiment, except for some manipulated aspects that were explained afterward. In the light of this explanation, subjects were asked to comment on whether their results could still be used in our study. All signed an *ex post* consent form.

The digital audio recordings and verbatim transcripts were encrypted.

## 3. Results

### 3.1. Characteristics of the sample

The sample consisted of 14 people (5 females, 9 males). Participants were between 22 and 35 years of age (mean = 26 years, SD = 3.73). Of them, 8 participants were in a relationship, 2 were married, and 4 were single. All participants had a university degree. The EBW group consisted of 3 females and 4 males; their mean age was 28 years (SD = 4.2). The LBW group, on the other hand, consisted of 2 females and 5 males, whose mean age was 24 years (SD = 1.96).

### 3.2. Results of the statistical analysis and the content analysis

**FIGURE 1:**
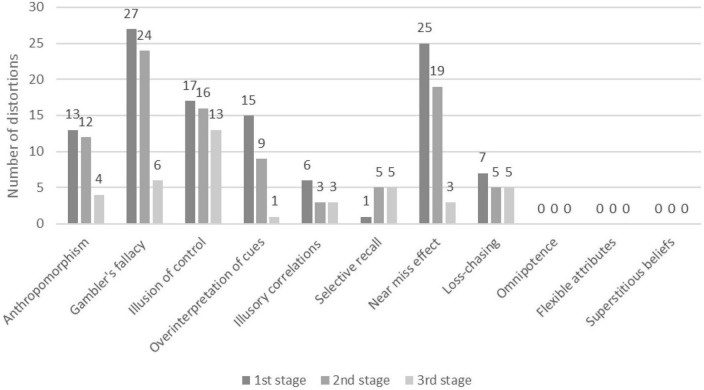
The number of individual cognitive distortions in the different stages of the simulation (in the whole sample).

Altogether 244 verbal units were identified as automatic thoughts biased by cognitive distortions. Considering the whole sample, the most common distortions were the Gambler’s fallacy (*n* = 57), Illusion of control (*n* = 46), and Near miss effect (*n* = 47). As presented in Figure 1, much larger numbers of cognitive distortions were found in the first (111 identified cognitive distortions) and second stages (93 identified cognitive distortions) of the simulation (stages of big wins), than in the last stage (40 identified cognitive distortions).

**FIGURE 2:**
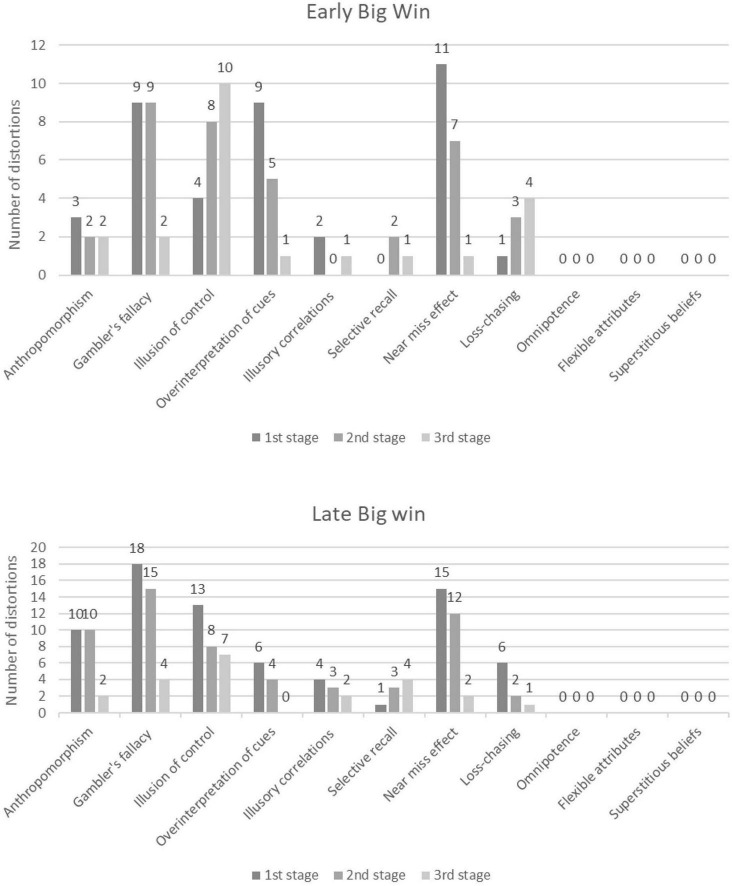
The numbers of individual cognitive distortions in the different stages of the simulation, separately for the Early Big Win Group and the Late Big Win Group.

Regarding the individual results for the two experimental groups, in the EBW Group altogether 97 verbal units, while in the LBW Group 152 verbal units were identified as distorted thoughts (Figure 2). Although the LBW group showed more cognitive distortions, and more distortions emerged in the first two stages of the study, there were no statistically significant differences between the two groups or between the individual stages [χ^2^(2,14) = 2.69, *p* = 0.260].

Despite the lack of statistically detectable difference, it is apparently worth reviewing our results qualitatively, as they can provide important information.

In the EBW Group, the Gambler’s fallacy (*n* = 20), Illusion of control (*n* = 22), and Near miss effect (*n* = 19) proved to be the most common distortions registrable in the subjects’ thoughts.

Similarly, in the LBW Group, the most common cognitive distortions were the Gambler’s fallacy (*n* = 37), Illusion of control (*n* = 28), and Near miss effect (*n* = 29). Anthropomorphism was also identified in large numbers in the LBW Group, but it is important to point out that out of the 22 identified contents 14 were found in the same person’s verbalizations.

More cognitive distortions emerged in the LBW Group during the first (*n* = 73) (without a big win) and second (*n* = 57) (with a big win) stages of simulation, compared to the EBW Group (first stage with a big win: *n* = 39; second stage without a big win: *n* = 36). Regarding the last stage (without a big win), a nearly identical moderate number of cognitive distortions were identified in the two groups (EBW Group: *n* = 22; LBW Group: *n* = 22). It is also important to emphasize the difference in the frequency of occurrence of the distortion Loss-chasing between the two groups. In the EBW Group, a greater number of wins came in the first stage of the simulation, and later – due to less frequent wins and more losses – subjects had less virtual credits available. The distorted thought of Loss-chasing was identified with increasing frequency in parallel with continuous losses (first stage: *n* = 1; second stage: *n* = 3, third stage: *n* = 4). The opposite phenomenon was found in the LBW Group. With the early losses the Loss-chasing effect was already there at the early stage of the simulation, while in later stages its occurrence become less frequent (first stage: *n* = 6; second stage: *n* = 2, third stage: *n* = 1).

Merely a few verbalizations were identified as manifestations of the cognitive distortions of Overinterpretation of cues (Σ*n* = 25; EBW Group: n = 15, LBW Group: *n* = 10), Illusory correlations (Σ*n* = 12; EBW Group: *n* = 3, LBW Group: *n* = 9), and Selective recall (Σ*n* = 11; EBW Group: *n* = 3, LBW Group: *n* = 8).

It is important to highlight that we did not find any contents indentifiable as manifestations of Omnipotence, Flexible attributes, or Superstitious beliefs in the groups.

## 4. Discussion

In line with previous research, our current study made use of the “thinking aloud” procedure (Toneatto, 1999; Joukhador et al., 2004) to study the cognitive distortions mirrored in the automatic thoughts of subjects while gambling. The verbal contents were studied with the help of qualitative content analysis, which gives a direct insight into people’s thinking from a broad perspective (Brown and Lenneberg, 1954).

Our primary research aim was to study whether subjects of a healthy sample show similar cognitive distortions to problem gamblers. According to our results, certain cognitive biases characteristic of gambling disorder (Griffiths, 1994; Toneatto et al., 1997; Goodie and Fortune, 2013) were present in our healthy sample as well. These results are consistent with previous studies that included members of the general population (e.g., Ciccarelli et al., 2017; Donati et al., 2018). The identified cognitive biases appeared quickly and in relatively large numbers in our study. This indicates that healthy members of the general population show automatic thoughts similar to cognitions of persons with a behavioral addiction to gambling.

It was the cognitive distortion of the Gambler’s fallacy that emerged most commonly in the verbalizations of our subjects. According to Kahneman and Tversky (1972), the gambler’s fallacy occurs because of representativeness heuristic, in which chance is perceived as a self-correcting process. Deviation in either direction causes a divergence in the other direction to restore balance. Representativeness heuristic describes the common systematic error we make when having to evaluate uncertain events, and the common error underlying Gambler’s fallacy may explain the high presence of this distortion in our sample.

Near-miss outcomes were not preprogrammed in our simulation as we attempted to study whether healthy persons also recognize near-misses. A large number of verbalizations point out that non-problem gamblers are sensitive to these events, in a way similar to problem gamblers. The findings of Habib and Dixon (2010) may explain our results. They suggest that problem gamblers tend to interpret near-misses as win-like events, whereas non-problem gamblers register them as well, but consider them a losing outcome.

In literature, the Illusion of control is considered to be a prominent sign of problem gambling (Langer, 1975; Cowley et al., 2015). We found several verbal contents identifiable *per definitionem* as the manifestation of the distortion of Illusion of control in the subjects’ thoughts. However, if we take a closer look at these contents, we can conclude that they were not signs of distorted reality perception. On the contrary, these verbal manifestations can be considered as attempts of the participants to understand the underlying mechanism of the simulated slot machine (e.g., “…*later I may be able to estimate after how many spins I should push stop so that I can win. Or I should test if I pressed stop when a grape is in the middle row of the first wheel, what the result would be. If it is consistently the same, then I could try for the other wheels as well. Then again, I may not find any form of regularity or a pattern./EBW group, participant no. 4./”*).

Anthropomorphism was registered in a relatively large number in the LBW Group. Anthropomorphism in its barest form is a pervasive, universal way of thinking, because it is an entity that is easy to grasp to create connections between humans and non-human agents (Mithen, 1996). Although this distortion was mostly found in one particular subject, its general, genuine human nature may explain its frequent occurrence.

An important finding of our study is that subjects have fewer cognitive distortions during the last stage of the simulation (third stage, without significant wins). This suggests that a further important difference between gamblers and non-gamblers can be that the latter is capable of correcting the appearing distorted thoughts in a quick and spontaneous manner.

Cognitive biases reflecting a severe distorted sense of reality and loss of control like superstitious beliefs (Joukhador et al., 2004), feeling of omnipotence (Rosenthal, 1986), or flexible attributes (Griffiths, 1994) did not emerge in the transcript. Our subjects did not produce any verbal manifestations identifiable as superstitious beliefs. These results are consistent with the outcomes of Joukhador et al. (2004), who found significantly less superstitious beliefs in non-problem gamblers compared to problem gamblers. In the case of gamblers, these superstitious beliefs usually appear in the form of behaviors and actions (Custer and Milt, 1985), which gamblers make attempts with to have an effect on the result of a bet (Rogers, 1998; Toneatto, 1999; Joukhador et al., 2003, 2004). These behaviors develop after a longer active gambling period (Joukhador et al., 2004), which also suggests that these cognitive distortions could indicate the presence of a gambling disorder or its severity.

No verbal contents reflecting that our subjects believe that they can play better than others or are all-powerful (Rosenthal, 1986) were identified. That is, the feeling of omnipotence was not present in the sample. Omnipotence can be motivating to achieve something, especially in the case of persons with gambling disorder who take it to extremities, which can interfere with the person’s ability to adhere to reality and set realistic goals (Selzer, 1992).

A further specific cognitive distortion not identified in our sample is Flexible attribution. In the case of flexible attributes, gamblers attribute their winnings to their own skills, and their losses to externals, which means that they heavily distort reality in a subjective manner and struggle with the loss of control (Griffiths, 1994).

Our second research aim was to study the effect of big wins on the occurrence of cognitive distortions in our sample without gambling-related problems. Research suggests that early big win plays a role in gambling behavior and the development of gambling-related cognitive distortions (Kassinove and Schare, 2001). Big win affects motivation to gamble and drives people to anticipate wins despite reality (Weatherly et al., 2004). To investigate whether the timing of big wins contributes to the emergence and characteristics of cognitive distortions, we created a group of early and a group of late big winners. We found a difference between the number of identified distorted thoughts in the two groups. More verbal contents of the LBW Group were interpreted as manifestations of cognitive distortions. The LBW Group experienced heavy losses in the first phase of the simulation, which may have generated frustration and higher tension in the participants, provoking cognitive dissonance in them. Therefore, members of the LBW Group might be more prone to using cognitive strategies to resolve the frustration in order to reduce cognitive dissonance (Festinger, 1962).

A further difference between the two groups is the incidence of Loss-chasing. According to Lesieur (1979), this cognitive distortion is one of the most important factors in the development of gambling. In the EBW Group, this phenomenon occurred in larger numbers in the later phases of the simulation. Participants expressed a desire to win back the assets they lost, and were motivated to possess the same amount of money as the highest they had achieved during the simulation or an even higher sum. In comparison, in the LBW Group Loss-chasing appeared in the earliest stage of the simulation, since the participants experienced losses as soon as they started the game, and they expressed that they would like to win back the amount they lost. This difference between the two groups illustrates that both a person’s losses and wins can be motivational in returning to gambling (Weatherly et al., 2004). Furthermore, losses and “big wins” can provoke different cognitive distortions, encouraging the person into further gambling behavior.

Our results suggest that the presence of cognitive distortions associated with the reduction of reality control and loss of control may be a risk factor for problem gambling.

### 4.1. Limitations

The main limitation of the study is the composition of the sample. All subjects have a university degree, which means that the study sample is positively biased in terms of the level of education.

One limitation of the experiment was that no subjects with potentially high-risk factors in their background, e.g., persons with low socioeconomic status and/or psychoactive substance use participated in the study. Moreover, personality traits characteristic of gambling and IQ were not assessed (Welte et al., 2004). Further studies including subjects with personal characteristics considered risk factors for the development of gambling could provide relevant information on the irrational thought processes of problem gamblers.

The subjects did not receive any real monetary winnings, which creates a different motivational environment from that of gambling and limits the level of simulation.

Another limitation is that even though the program used in the simulation mimics quite well the earlier versions of slot machines, it lacks certain aspects of modern virtual slot machines, e.g., visual and sound effects.

Moreover, it is important to note that Ramnero et al. (2019) point out that studying the effects of a “big win” in a limited experimental design can be difficult. This statement highlights a further limitation of our study. The characteristics of the environment outside the computerized game (i.e., gambling-related cues as the room features such as noise, lights, and colors) could not be provided in our university office, making it less able to simulate the conditions of real slot arenas.

## 5. Conclusion

Our qualitative analysis identified in the automatic thoughts of healthy subjects the presence of cognitive distortions typical of problem gamblers, but we did not find cognitive distortions suggesting a significant loss of control or a distorted sense of reality. Accordingly, the appearance of reality-checking uncertainty or loss of control can be alarming for the development of gambling. Moreover, early big wins lead to a more intense loss-chasing, while late big wins provoke frustration handled by distorting the interpretation of the situation.

## Data availability statement

The original contributions presented in this study are included in the article/Supplementary material, further inquiries can be directed to the corresponding author.

## Ethics statement

The studies involving human participants were reviewed and approved by the Regional and Institutional Scientific Research Committee of the University of Debrecen (approval number DE RKEB/IKEB: 5641-2021). The patients/participants provided their written informed consent to participate in this study.

## Author contributions

RK established study concept and design, analyzed and interpreted qualitative data, carried out statistical calculations, and took part in the preparation of the manuscript. DÖ analyzed qualitative data and took part in the preparation of the manuscript. ZF analyzed qualitative data, took part in the preparation of the manuscript, and supervised the study and the publishing process. All authors had full access to all data in the study and took responsibility for the integrity of the data and the accuracy of the analysis.
