# Supplementary material for: Non-problem gamblers show the same cognitive distortions while playing slot machines as problem gamblers, with no loss of control and reduced reality control, though – An experimental study on gambling
Source: Front Psychol. 2023 May 22;14:1175621. doi: 10.3389/fpsyg.2023.1175621 (PMC10239866; doi:10.3389/fpsyg.2023.1175621)
Supplement: Supplementary file 1 [file Data_Sheet_1.docx]

*Supplementary Material*

**Non-problem gamblers show the same cognitive distortions while playing slot machines as problem gamblers, with no loss of control and reduced reality control, though - An experimental study on gambling**

**Róbert Krébesz, Dóra Kata Ötvös, Zita Fekete^*^**

***Correspondance:** [fekete.zita@med.unideb.hu](mailto:fekete.zita@med.unideb.hu)

**Supplement A**

*Instructions of the slot machine simulation applied in the study*

Now, I will ask you to play three sets of the well-known "fruit" game. The game works the same way as a traditional slot machine. You can start the spins by using the mouse or the SPACE button. You should also use the STOP button if you want to stop the spins early, again by using the mouse or pressing ENTER.

Also, I would like to ask you to tell me your thoughts and talk about the feelings and experiences you are having while playing. You can say anything that comes to your mind. You can think of a variety of things. There are no good or bad thoughts, so many people, so many minds. In the meantime, I'm going to record everything on a tape recorder. This is only necessary for me not to forget anything. The recording is for my work, noone else will have access to it.

At the start of the game, you can roll out a starting amount to play with during the fruit game. One bet costs 100 virtual credits. At the beginning of the game, the multipliers for each symbol are shown, i.e., if you spin a full line of that symbol, it will increase the bet amount by a multiple of the amount you have played.

The whole game takes about 30 to 50 minutes. Thank you for participating!

**Supplement B**

*Detailed description of the slot machine simulation (Titled: Bedinu C)*

- The simulation started with the players having to read 4 shortpages on the instructions, in which subjects were asked to verbalize their thoughts and feelings while playing.
- Following the instructions, subjects were presented with a wheel of fortune (Figure 1/A), which apparently chose an initial stake at random. In order to control the test, the wheel was programmed in a way that each participant spinned a starting stake of 5,000 credits.
- After having been presented the wheel of fortune, subjects met an interface showing the symbols used in the simulated slot machine game and their corresponding multipliers, which were presented continuously throughout the rest of the game (Figure 1/B).
- After that, subjects played 90 spins (the simulation consisted of three sets with 30 spins/set). The visual design of the slot machine program emulated real life online machines, displaying the symbol wheels, buttons and a pay table. Every bet cost a constant of 100 credits; participants were not allowed to change that. Subjects also had the option to stop a spin. If not stopped prematurely, every spin lasted 20 seconds, which gave the participants enough time to verbalize their thoughts and feelings. This interface displayed 3 wheels of symbols that simulated the operation of slot machines (Figure 1/C). The wheels spinned in a 3x3 matrix displaying 9 symbols after stopping. In the game, a spin was a winning one if there were 3 identical symbols in the middle row of the matrix after the spin stopped. When a spin resulted in a win, the program played a sound effect and displayed a sign. After such a win, the program displayed the amount just won in a flashing window, which disappeared within moments. Furthermore, the program continuously displayed the amount of credits the test subject currently owned. Like in the case of the wheel of fortune, the 90 spins that subjects played during the experiment had preprogrammed outcomes. This ensured that subjects participate in the study under the same conditions.

Credit


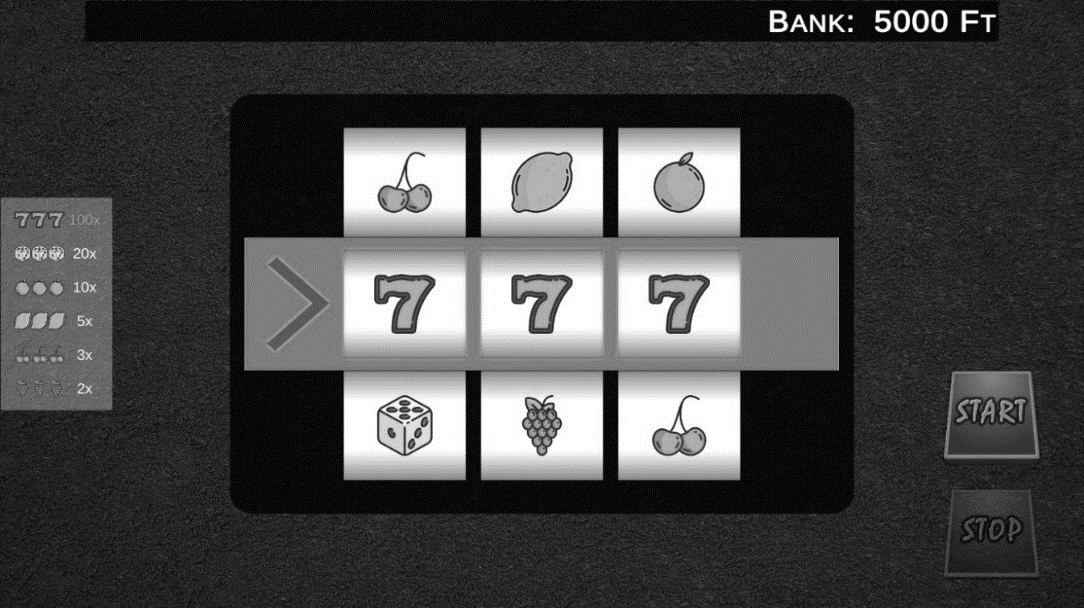

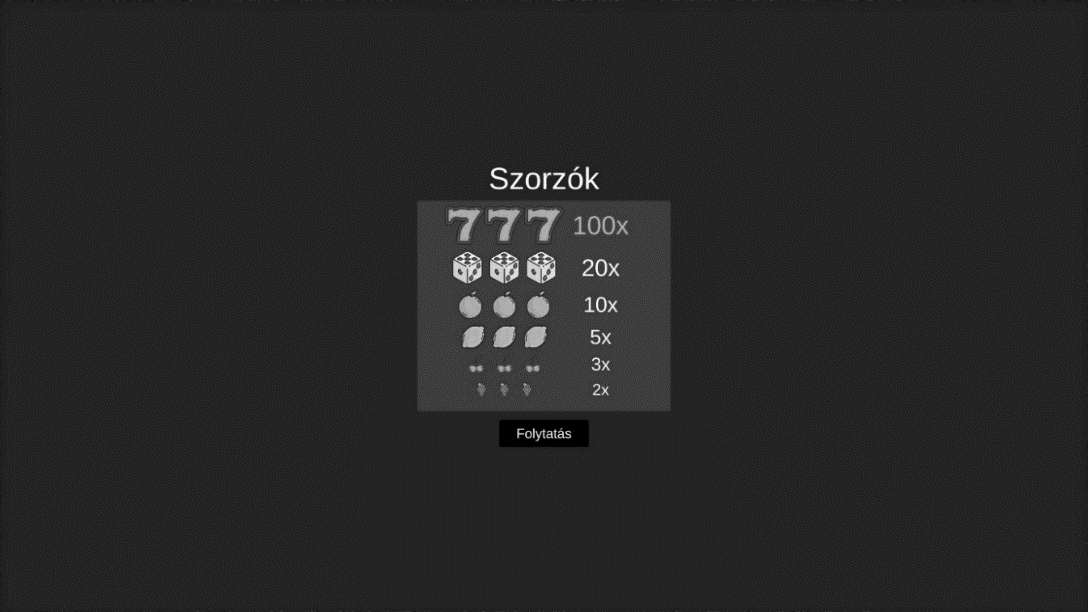

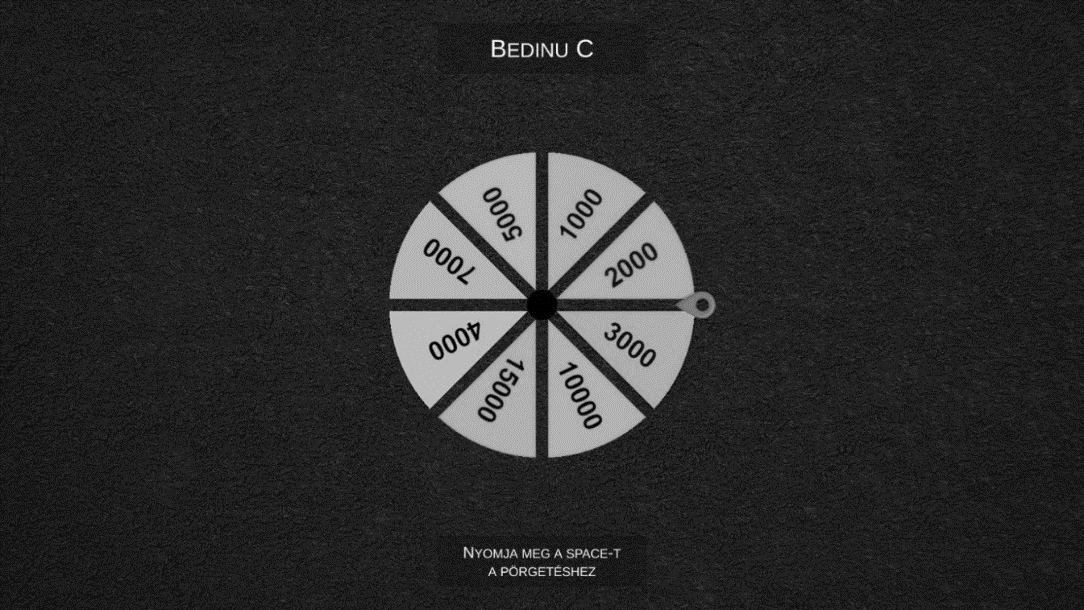


Figure 1. The slot machine simulation (A: Wheel of fortune, B: Multipliers,

C: Graphical interface of the simulated slot machine)

C

B

Continue

Multipliers

Press SPACE to spin the wheel

A

**Supplement C**

*Definitions of cognitive distortions in gambling used in the study as a coding matrix, with examples from the transcripts*

| Cognitive distortions coded in the transcripts | Definition and examples emerged in the transcripts |
| --- | --- |
| *Anthropomorphism* | Attributing human, living characteristics or talking to the slot machine.  e.g,, *"Oh, you cheater!", "It won’t let me win."* |
| *Gambler’s fallacy* | Attempts to come to a conclusion about the next spin, based on previous experience. Expressing beliefs that the outcome of a bet can be predicted based on previous experience.  e.g,, *"I will win again... I would like to say it was a hunch, but is it possible that it wins every fifth round? Because now… the wins seem relatively consistent I guess, I will start to count the spins from now. "* |
| *Illusion of control* | Expressing ideas involving that the player can influence the outcome of a bet by using certain knowledge or skills. Ideas of cause and effect arise during the simulation.  e.g., *"I think I can control this a little bit… the result of a bet. I control it a little bit… the symbols stop so slowly, not even close to when I want them to. The second and third symbols stop even later than the first. So I barely have any influence on it, in my opinion. The worst thing is that it feels good to win a bet, especially if it is a large sum, but that is what this is about. It is bad, because I did not win this because of me, instead the program generated the result. "* |
| *Omnipotence* | Expressing ideas that the player is better at slot-machine gambling than others. Thoughts of having power over the outcome of a bet.  *No such occurrence emerged in the text.* |
| *Overinterpretation of cues* | Referencing otherwise inadequate stimuli as decisive factors during the slot machine gambling. Mentioning mental or bodily states that could influence the outcome of a bet.  e.g., *"I feel like cheering only has a moderate effect, because I will not get the credits at the end.", "Grapes, grapes, grapes, come on grapes… Oh why didn’t it stop!?"* |
| *Illusory correlations* | Thoughts about certain external stimuli that could potentially influence the outcome of a bet.  *No such occurrence emerged in the text.* |
| *Flexible attributes* | Attributing different causes for wins and losses. Ideas that a win occurs because of skill or behavior, and losses occur because of external circumstances.  e.g., *"If someone notices a rule or pattern, for example memorizing the order the symbols appear on a wheel, they may influence the outcome, instead of letting lose it. The end result is different if I stop it at the right time."* |
| *Selective recall* | Maximizing wins and minimizing losses during slot machine gambling. Expressing difficulties recalling losses.  e.g., *"I didn’t notice how many bets I made, that my balance is so low. Or I didn’t win as large amounts as I thought. "* |
| *Superstitious beliefs and rituals* | Mentioning or verbalizing beliefs that suggest the subject could potentially influence in a superstitious way the outcome of bets. Performing ritualistic behaviors to influence the outcome of bets.  *No such occurrence emerged in the text.* |
| *Loss-chasing* | Expressing ideas to get "even" during slot machine gambling. Mentioning thoughts about winning back the lost amount and verbalizing losses.  e.g., *"My goal now is to get back to my starting balance."* |
| A further cognitive phenomenon coded | |
| *Near miss effect* | Expressing ideas and verbalizing thoughts that a lost bet was *"*near/close*"* to a winning state.  e.g,, *"I thought it really would be a grape. I was hoping for it so much. Two of them were there and then…", "It would be better if all of them were cubes. So close. ", "My heartrate increased… One lemon, then two lemons and… almost! "* |
